# Developmental Approaches to Chronic Pain: A Narrative Review

**DOI:** 10.7759/cureus.45238

**Published:** 2023-09-14

**Authors:** Nikita P Patel, Chad M Bates, Aakash Patel

**Affiliations:** 1 College of Medicine, Nova Southeastern University Dr. Kiran C. Patel College of Osteopathic Medicine, Davie, USA; 2 Anesthesiology, Albert Einstein College of Medicine, Jacobi Medical Center, Bronx, USA

**Keywords:** pain modulation, opioid use, alternative medical therapies, opioid alternative, chronic pain management

## Abstract

Chronic pain, which can potentially develop from acute pain, subacute pain, or breakthrough pain, is generally defined as pain persisting for greater than three months with minimal relief. Chronic pain can be associated with a myriad of medical conditions. It is also one of the most common causes of disability, physical suffering, depression, and reduced quality of life. Treatment can vary depending on the underlying pathophysiology and can involve physical therapy, non-pharmaceutical approaches, pharmaceutical drugs, and invasive procedures. Currently available pharmaceutical agents have been effective for short-term management of chronic pain conditions, but few options address chronic pain with long-term efficacy. First-line pharmaceutical agents can potentially include over-the-counter (OTC) or prescription-strength non-steroidal anti-inflammatory drugs (NSAIDs), which have been linked to numerous side effects. If chronic pain persists, steroids are frequently used to provide longer relief. For more progressive or resistant chronic pain and/or in conjunction with invasive procedures, opioids have been utilized for acute treatment and for long-term maintenance. While these agents have proven to be effective for both acute and long-term use due to their modulation at various peripheral and central opioid receptors, they can be associated with numerous side effects and tied to the risk of addiction. As such, an unmet need exists to identify treatment modalities that provide opioid-like pain relief without opioid-induced adverse effects and the potential for addiction. This narrative review will provide an overview of the currently available treatment modalities for chronic pain and their adverse event profiles, as well as a review of therapies that are currently in development and/or preclinical trials for the management and treatment of chronic pain.

## Introduction and background

Pain is one of the most common conditions faced by many people in society. According to the 2019 National Health Interview Survey, researchers found that chronic pain affects approximately 20.5% of the American population [1]. In addition to being common, pain is also a distressing experience that results in a change in an individual’s physiological state. The duration of pain can be further classified into acute, subacute, chronic, and breakthrough pain following the intake of analgesics. Acute pain develops suddenly as a result of injury, infection, and/or bodily disequilibrium, such as decompensated end-organ dysfunction. Acute pain, although sometimes pathologic, can be thought of as an evolutionary adaptive mechanism that shields the body from further harm. Contrary to acute pain, chronic pain is a pathological development and persists for several months with minimal relief [2]. Acute pain is postulated to be a primitive and adaptive response developed over time for survival, evolution, and emotional associations, whereas chronic pain is less helpful and poses a significant problem and burden to the healthcare system, economy, and society. While pain can be adaptive and provide signaling for alarm or protection, it can also become maladaptive and chronic due to a myriad of genetic and environmental factors. This can lead to a transformation from acute to chronic pain. Current treatment options aim to reduce pain by interfering with the transmission signal at all levels of pain processing. This mechanism of interference includes peripheral afferent pain-sensing neurons, the ascending spinothalamic tract, the thalamus, and the primary somatosensory cortex. This multilevel pain processing pathway provides targeted points for analgesics to work on. 

Before discussing the various analgesics that are commonly used for pain, it is important to understand not only how pain is processed but also its development from acute to chronic. The painful process is thought to begin with a primary persistent insult resulting in up-regulation of cyclooxygenase 2 (COX-2) and IL-1b [2]. This up-regulation sensitizes first- and second-order spinal neurons, resulting in altered neuronal structure and function [2]. This sensitization of primary and secondary neurons by proinflammatory cytokines can result in a decreased activation threshold, increased sensitivity, and persistent signaling throughout the peripheral and central nervous systems [2].

NSAIDs are becoming increasingly popular for temporary acute pain relief, as they can be obtained without a prescription and are found over-the-counter (OTC). NSAIDs are a class of medication that functions as non-selective inhibitors of cyclo-oxygenase 1 and 2, which prevent the generation of prostaglandins, thromboxanes, and prostacyclins [3]. The inhibition of these proinflammatory enzymes reduces inflammation, redness, tenderness, and edema at the injured site [3]. However, given that this relief centers around the inhibition of COX1 and COX2 enzymes, therapeutic analgesia is only effective for the duration of COX1 and COX2 lifespans. Therefore, the synthesis of additional COX1 and COX2 enzymes will reinstate the pain cycle. Thus, NSAIDs provide only a temporary solution to pain and may pose multiple adverse effects with long-term use. Side effects associated with long-term use include gastrointestinal (GI) ulcers, hepatotoxicity, nephrotoxicity, increased cerebrovascular accident risk, and pulmonary inflammation/irritation [4]. Given these long-term effects of chronic use, NSAIDs are only able to provide safe short-term therapeutics when indicated.

Steroids are a class of medications that are frequently used to manage chronic pain. Steroids such as dexamethasone, prednisolone, hydrocortisone, triamcinolone, and methylprednisolone are available either orally or via injection. Steroids (which are lipophilic) enter the cell, bind to an intracellular protein or carrier, and enter the nucleus to bind to DNA and alter the transcription of various genes. Although each steroid uniquely alters gene expression, after entering the nucleus, the most common mechanism of action is inhibition of pro-inflammatory cytokines (i.e., interleukin 1B, interleukin 2, tumor necrosis factor-alpha, et al.) and chemokine gene transcription [5]. The glucocorticoid prednisone, upon entering the cell, forms a glucocorticoid-receptor complex that binds to DNA and inhibits the transcription of various proinflammatory cytokines [5]. Cytokines are released during periods of chronic and acute inflammation and are partially responsible for the transmission of pain signals from visceral structures to the CNS. Therefore, the use of steroids diminishes the afferent pain signals to the brain and, consequently, the perception of pain. However, steroids are only moderately effective at treating chronic pain, partially due to the potential adverse effects. Steroids are known to contribute to immunosuppression, the development of insulin resistance, growth suppression, osteoporosis, cardiovascular impairment, and impaired wound healing [6]. These side effects are significant because, although symptomatic relief is provided, steroids fail to treat the underlying condition and can potentially introduce additional co-morbid conditions. Given these adverse effects, steroids only pose a temporary solution to a chronic problem. Therefore, additional and more effective chronic pain modulation is needed.

Another pharmaceutical approach includes the use of opioids such as morphine, codeine, and oxycodone, which target opioid receptors to reduce pain and diminish the pain incident [7]. Therefore, opioids are commonly prescribed for managing both acute and/or intractable pain [7]. Unfortunately, however, opioid-induced mortality has increased by more than fourfold since 1999 [8], resulting in an epidemic. The medical consequences of abusive and/or chronic opioid use can include tolerance, psychological addiction, physical dependence, increased pain sensitivity, and a potentially increased risk for neuropsychiatric disorders such as anxiety disorder, organ dysfunction, and mortality. Given these potentially fatal side effects, current investigations are ongoing about potential developmental approaches to the treatment of acute and chronic pain.

Although not a first-line agent for chronic pain, tricyclic antidepressants (TCAs) and serotonin noradrenaline reuptake inhibitors (SNRIs) can occasionally be repurposed for the treatment of chronic pain. These medications can potentially serve as an adjuvant to neuropathic pain. SNRIs function centrally by inhibiting the presynaptic uptake of serotonin and noradrenaline, thereby increasing the synaptic concentration of these neurotransmitters. The increased concentration of noradrenaline in the CNS has been shown to provide analgesia through modulation of ɑ2 adrenergic receptors located on the spinal cord’s dorsal horn [9]. Noradrenaline binding to ɑ2 adrenergic receptors promotes neuronal hyperpolarization by enhancing post-synaptic potassium influx and reducing presynaptic calcium influx [9]. The resultant hyperpolarization of neurons in the spinal dorsal horn can potentially diminish the propagation of pain signals centrally responsible for pain perception. However, the usage of these medications to treat chronic pain is limited because of the potential for drug-drug interactions, discontinuation syndrome, and/or adverse events.

Lastly, refractory pain that is unresponsive or minimally responsive to the above-mentioned treatment options is occasionally recommended to undergo invasive and/or minimally invasive procedures. These procedures include, but are not limited to, medial branch blocks, medial branch nerve ablations, and spinal cord stimulation. These various procedures are becoming more popular as they offer more selective targeting of chronic pain-producing structures than their NSAIDs and opioid counterparts. Additionally, invasive and minimally invasive procedures can potentially provide a longer duration of analgesia with a single treatment. Although these methods of targeting chronic pain are more selective than others on the market, they pose side effects such as potential infection, patient discomfort, hormonal imbalance, and tolerance.

With all the adverse effects mentioned above, treatment options like NSAIDs, opioids, steroids, TCAs, gabapentin, pregabalin, capsaicin, cannabinoids, and SNRIs fail to provide a long-term, efficacious, and safe solution to chronic pain management. In addition, invasive procedures are also not always effective and are occasionally associated with adverse events. Thus, an unmet need in the management of chronic pain persists. As such, research efforts are evaluating more targeted developmental pharmaceutical agents for the treatment of acute and chronic pain. Ideally, these explorations will identify potential developmental approaches that still produce the desired opioid analgesic effects while minimizing adverse effects. Although these developmental approaches are each unique and will be discussed separately, they all center around a similar mechanism of stimulation/modulation of opioid receptors. This stimulation/modulation of opioid receptors mimics the effects induced by opioids without the need for exogenous administration of opioids. This narrative review aims to provide an overview of the currently available literature on developmental approaches to chronic pain management. Although there are many non-pharmacologic and herbal therapies used to treat chronic pain, such as yoga, acupuncture, medical cannabinoids, and capsaicin, this narrative review aims to focus on the pharmacologic developmental approaches to chronic pain. 

## Review

Delta-opioid receptor agonist

The delta-opioid receptor (DOR) belongs to a class of G-protein-coupled receptors that binds to and is stimulated by opioid analgesics. Although there are no currently available DOR agonists, there is significant research currently in development that centers around modulation of the DOR in order to provide therapeutic analgesia. Thus, the DOR is one of the most commonly studied targets for pain management due to its wide distribution in the brain and central nervous system (CNS). More specifically, it is located in the ascending and descending pain pathways, periaqueductal gray area (PAG), rostral ventromedial medulla (RVM), cerebral cortex, and amygdala [10]. The DOR being highly distributed throughout pain-modulating areas holds potential to affect multiple pain-processing structures. This potential multitargeted approach can ideally allow for greater potential efficacy. In addition, because of the DOR’s location in the emotion processing/association center [10], the amygdala and agonism of the DOR can potentially offer dual therapy. This dual therapy action of delta-opioid receptor agonists on the amygdala has the potential to modulate pain and the consequent emotional association, similar to currently available opioid therapy. As such, one of the proposed mechanisms of pain modulation is targeting intracellular pathways such as G-protein coupled receptor (GPCR) kinases, LIM kinases, and regulator of G-protein signaling 4 (RGS4) to increase DOR membrane expression [10]. 

LIM Kinase 

LIM kinase is an intracellular molecule that modulates the expression of neuronal DORs. Through phosphorylation of protein cofilin, which functions to bind and aid microfilament actin cytoskeletal transport, LIM kinase can inhibit intracellular cytoskeletal transport of proteins, including the DOR [10]. One of the proposed methods to increase DOR membrane concentration is through inhibition of LIM kinase. LIM kinase inhibition thereby removes the inhibition on DOR membrane transport and subsequently increases the concentration of DOR membrane expression levels [10]. This developmental approach holds promise to potentially target a single enzyme, which is something that has yet to be achieved or utilized. In addition, LIM kinase inhibition may potentially decrease the rate of unwanted side effects and reduce tolerance to opioids. This is thought to be attributed to the selective targeting of a single enzyme and decreasing the modulation of other cellular processes [10]. Lastly, complementing the selectivity of LIM kinase inhibitors, dual therapy with other pain-modulating agents has the potential to provide multilevel analgesia.

RGS4 Protein Modulation

The last method currently being studied for increasing DOR cell membrane expression is through modulation of RGS4. RGS4 is a protein that catalyzes GTP hydrolysis on various GPCRs, namely GTP-bound Gα. This increased rate of GPCR inactivation was previously thought to halt the intracellular signal propagation of resultant analgesia upon opioid binding. Numerous small RGS4 inhibitors have been and are currently in development, including cysteine covalent modifiers [11] and peptide-based RGS inhibitors [12]. These modifiers and inhibitors work by inhibiting the GTPase action of RGS molecules, which allows for an increased duration of GPCR activity and downstream signaling [11,12]. In the context of pain, where the body’s homeostatic mechanism naturally produces endogenous opioids [13], the increased duration of GPCR signaling potentially affords increased efficacy and duration of endogenous opioids. This increased duration and efficacy of endogenous opioids simultaneously lowers the unwanted side effects of exogenous opioids, including addiction, tolerance, respiratory depression, constipation, and death. Although clinical trials are not currently underway, research efforts on in-vitro yeast yielded isolates of RGS4 inhibitor peptides [14]. While these current studies hold potential promise, additional research is needed to determine the efficacy, long-term safety profile, and durability of this treatment modality.

G-Protein Coupled Receptors 

G-protein-coupled receptors (GPCRs) are a group of plasma membrane receptors that bind various ligands, including opioids. Upon binding its respective ligand, GTP is hydrolyzed into GDP, leading to the dissociation of the beta and gamma GPCR subunits and the subsequent activation of downstream signaling [13]. GPCR signaling is inactivated by phosphorylation via G-protein receptor-coupled kinases (GRKs) and subsequent binding via arrestins. Given that G-protein-coupled receptors (GPCRs) regulate numerous cellular processes and can amplify downstream effects, they are another potential target for increasing the expression of neuronal DOR. In vitro studies on cellular and wild-type neurons show that upon activation of GPCRs and the resultant phosphorylation cascade, there is unavoidable phosphorylation of DORs, recruitment of B-arr1, and consequent DOR degradation via clathrin-coated pits [10]. Utilizing this method of targeting GPCRs by inhibiting the activation of GPCRs, the resultant amplification cascade is halted. This allows significantly more DORs to be trafficked to the plasma membrane [10]. Among all the therapies used to increase DOR plasma membrane concentration, this is by far the most unique because it is the only therapy that halts the generation of an intracellular amplification signal. The selective targeting of an extracellular protein or structure is beneficial as intracellular protein targets (LIM-kinase and RGS4) differ in expression among cell types, preventing a wide range of treatment options. This model of pain modulation is potentially the most potent in terms of increasing DOR plasma membrane concentration. Although preclinical studies are not currently being conducted, the findings of this study pose promise for preclinical studies evaluating GPCR inhibitors. Lastly, additional preclinical studies and further research are warranted to evaluate the potential cellular dysfunction that may ensue by halting a cell's signal transduction pathway.

Interleukin 1βR modulation

Although DOR modulation can potentially relieve pain through multiple mechanisms, another avenue to modulate pain that is currently being studied is IL-1βR modulation. IL-1 is a chemokine/cytokine that is released by a variety of cell types, such as macrophages, endothelial, epithelial, and microglial cells, in response to proinflammatory states such as injury, infection, or bodily stress. In addition, previously conducted animal studies found that peripheral sciatic nerve injury was associated with an increased concentration of P450c17 mRNA levels and P450c17 protein levels, a key enzyme needed for corticosteroid synthesis and a potential contributor to the development of neuropathic pain [15]. Given that IL-1 and P450c17 are known regulators of pain, alterations in either the expression or action of P450c17 or IL-1 are potential avenues to pain modulation. In a preclinical study performed using mice with induced sciatic nerve injury, it was found that IL-1b expression markedly increased during the first two days of injury, resulting in a corresponding decrease in P450c17 expression [16]. This indicates a negative correlative relationship between the two variables. These results indicate that stimulation of extracellular IL-1b cytokine receptors can potentially provide an analgesic effect through a decrease in intracellular pro-inflammatory signal cascades [16]. Although still debatable and preliminary, selective targeting of IL-1b in the spinal cord dorsal horn, brain parenchyma, peripheral nerve endings, and organ tissues can potentially produce the desired anti-nociceptive state. Due to the inverse correlation between the proinflammatory cytokine IL-1b and the protein P450c17, as found in this study [16], additional preclinical trials are underway to target receptors for IL-4 and IL-10.

Granulocyte-colony stimulating factor

The innate immune system is another area of research being explored to reduce pain perception. The innate immune system, in response to a bodily insult, recruits many cell types, including neutrophils, which secrete endogenous opioids such as b-endorphin (END), met-enkephalin (ENK), and dynorphin-A (DYN) [17]. Granulocyte-colony stimulating factor (G-CSF) agents are often used in injuries, systemic diseases, and chronic conditions to modulate the response of neutrophils and their innate healing properties. In preclinical studies, researchers used a xenograft oral cancer mouse model to determine whether exogenous administration of recombinant granulocyte colony-stimulating factor (rG-CSF) resulted in an increase in neutrophil infiltration [18]. This increase in neutrophil migration can potentially increase the secretion of endogenous opioids, which, over time, can lead to a delayed analgesic effect. [18]. The researchers found that treatment with rG-CSF increased opioid-containing Ly6G+ neutrophils in both male and female mouse models [18]. In addition, the increase in lymphocyte antigen 6 family member G (Ly6G+) neutrophils was accompanied by a concurrent decrease in gnawing, a sign of orofacial carcinoma-induced nociception [18]. These results explore pain modulation with respect to the differing male and female physiologic and emotional responses to pain. Based on the finding that systemic administration of rG-CSF resulted in a reduction in nociceptive signaling [18], direct injection of rG-CSF into injured tissues, organs, nerve roots, and other structures is currently being evaluated. These direct injections, although still under investigation, could potentially provide a localized response through the infiltration of endogenous opioid-secreting neutrophils.

TIMP-1 and MMP modulation

During acute injury, the release of reactive oxygen species (ROS) as a result of tissue injury or death stimulates the regeneration and healing process, in which matrix metalloproteinases (MMPs) play a significant role [19]. MMPs are known to stimulate the inflammatory response by promoting cleavage and rearrangement of extracellular proteins, chemokines, and cytokines [19]. Consequently, this provides an environment for cellular cytoskeletal rearrangement and healing [19]. Although MMPs are proinflammatory and are implicated in many chronic pain syndromes, tissue inhibitor of metalloproteinases-1 (TIMP-1) offers regulation and inhibition of MMP to prevent pathological overactivation. As such, this is another potential target for pain modulation. Preclinical mouse studies found that TIMP-1 knock-out (KO) mice showed signs of mechanical hypersensitivity that were significantly reduced upon exogenous administration of recombinant murinerm TIMP-1 (rmTIMP-1), which was shown to halt the progression of inflammatory pain [20]. The results of this study may potentially be able to provide pain relief through multiple signaling pathways. By targeting TIMP-1, MMP over-activation and intracellular signal propagation can be modulated [20]. This regulation of pain signal propagation allows a multi-system approach to target pain, thus allowing for greater and more diverse treatment options for pain. Additionally, this preclinical study [20] highlights the potential for local injection of rmTIMP-1 to provide negative regulation for MMP-mediated inflammatory pain. However, further research is needed to further ascertain the efficacy, safety, and potential long-term side effects.

Sodium channel modulators

Local anesthetics, such as lidocaine, are often used to manage pain as they inhibit neuronal sodium channels (Na+) and the subsequent propagation of sensory signals to the central nervous system (CNS). However, these agents are limited in use and efficacy because they are administered locally to circumvent toxicity. Therefore, further research is aimed at alternative treatment options that can be used systemically to more effectively manage chronic pain. In preclinical studies using mouse models, knock-out (KO) deletion of Na+ 1.7 and Na+ 1.8 channels produced a significant loss of inflammatory pain [21]. This relationship between pain and Na+ channel excitability is not only confined to neuronal pain but is also clinically relevant as an inherited familial condition. Inherited erythromelalgia (IEM), a lifelong condition with intermittent pain attacks based on molecular studies, is associated with peripheral Na+ channel hyperexcitability [22]. Some small molecules and toxins are currently in clinical trials, such as Pfizer's selective aryl sulfonamide, pyrrolidine-based compounds, tetrodotoxin, protoxin II, and µ-Conotoxin-KIIIA [22]. This method to target chronic pain could be effective because each small molecule or toxin selectively inhibits a class of sodium channels [22]. Given that each subtype of Na+ channels- Na+ 1.7 and Na+ 1.8- is associated with different pathologies and is found in different bodily locations, selective inhibition allows for targeted therapies without systemic effects. Further research is needed to assess efficacy, long-term benefits, and potential side effects.

Interleukin-17 regulation 

Another avenue to target pain's effects involves the use of specific T-cell classes to modulate or facilitate pain. Based on preclinical studies performed on mice, Th1 and Th17 cells propagate an increase in IL-17 cytokine release, macrophage infiltration into chronic sciatic nerve injury sites, and macrophage accumulation in the dorsal root ganglion (DRG) [23]. This potentiation of pain is further supported by a pre-clinical study on bone cancer mouse models where IL-17 inhibition reduced mechanical allodynia and paw flinches, a sign of pain [24]. The release of this cytokine poses a potential target for pain modulation, as IL-17 cytokines are proinflammatory and recruit macrophages to sites of injury. As numerous pro-inflammatory cytokines are released during periods of inflammation, such as TNFα and IFNγ [24], targeting IL-17 is potentially effective as IL-17 antagonism eliminates the downstream signaling and activation of pro-inflammatory signals. Therefore, selective targeting of IL-17 could potentially serve to eliminate the generation of some pro-inflammatory signals. 

Leukocyte elastase inhibition

Additionally, nerve injury-resulting T-cell infiltration in the DRG releases leukocyte elastase (LE)-encoded by Elane, which activates proinflammatory MMP9 and results in the propagation of pain signals in the CNS [25]. The inhibition of leukocyte elastase (LE) is yet another potential target for pain regulation. To determine the extent of T-cell involvement in pain production, T-cells were harvested from wild-type (WT) mice and Elane -/- mice to determine the correlation between LE production and pain [25]. In this study, the researchers found that Elane -/- mice and SerpinA3N-treated mice showed a significant reduction in the development of neuropathic pain [25]. This particular developmental approach is unique from the other developmental approaches discussed in this review, as it utilizes a cDNA vector expressing SerpinA3N [25]. The results of this study open the door for future research in gene therapy-based pain treatment. 

Fingolimod (FTY720)

Another approach involves inhibiting CNS T-cell infiltration by utilizing the blood-brain barrier (BBB) permeable drug Fingolimod (FTY720) [26]. Studies in mouse models following partial sciatic nerve ligation showed that treatment with FTY720 resulted in decreased T-cell CNS migration, T-cell sequestration in lymph nodes, and significantly decreased mechanical and thermal pain sensitivity [26]. As Fingolimod is FDA-approved for the treatment of relapsing and remitting multiple sclerosis (MS), this drug can potentially have the ability to be used as a dual therapy in patients with MS and chronic pain. The ability of Fingolimod to modulate pain at the level of the CNS is unique in that there is less incidence of peripheral side effects, it has the potential to act directly on the pain processing and association centers, and it is more potent due to its direct effects on the CNS. Treatment utilizing Fingolimod (FTY720) is currently in preclinical trials as a monotherapy for chronic pain.

Glatiramer acetate (GA)

T-cells can be grouped into many different classes, including Th1, Th2, Th17, Treg, cytotoxic T-cells, and CD8+ regulatory T-cells. Each of these T-cells relies on particular cytokine signals that regulate the differentiation into each T-cell sub-group [27]. This T-cell regulation signal can be utilized to convert pro-inflammatory T-cells into anti-inflammatory T-cells. The conversion of pro-inflammatory T-cells to anti-inflammatory T-cells is complex and involves alterations in T-cell programming and cytokine production. One method that is under investigation involves the use of glutamate acetate (GA). Glatiramer acetate (GA) was used in preclinical studies on neuropathic and inflammatory mouse models and showed reduced microglial activation, decreased allodynia, and an increased number of dorsal horn CD4+ T-cells producing IL-10 [27]. This is beneficial because it is one of the only therapies that can reprogram the immune system at the level of the CNS. Table 1 shows the summary of results.

**Table 1:** Summary of results

Developmental Approach Discussed	Locations Found	Targets Studied	Mechanism of Action
Delta Opioid Receptor (DOR) Modulation	Amygdala, periaqueductal gray area, rostral ventromedial medulla, and cerebral cortex	CNS	Found to activate opioid receptors and modulates pain
LIM Kinase Inhibition	Chromosome 7	Cytoskeletal transporters	Modulates the expression of neuronal DOR’s via phosphorylation of protein cofilin
RGS4 Modulation	Cytoplasm	GPCR’s	Catalyzes GTP hydrolysis on GPCR’s leading to inactivation
G-Protein Coupled Receptors	Cell Membranes	DOR’s	Found to regulate cellular processes, amplify downstream signaling, and modulate DOR expression
Interleukin 1BR Modulation	Spinal cord dorsal horn brain parenchyma, peripheral nerve endings, and organ tissues	T-cells	Found to decreases P450c17 expression leading to pain modulation
Granulocyte Colony Stimulating Factor	Bone marrow	injured tissues, organs, and nerve roots	Found to modulate neutrophil recruitment and endogenous opioid secretion
TIMP-1 and MMP Modulation	Chromosome 11	Cytokines, proteins, and chemokines	Found to modulate the inflammatory response and rearrangement of extracellular elements
Sodium Channel Inhibition	Throughout the body	CNS	Found to inhibit sodium channel conduction and the propagation of sensory signals
Interleukin 17 Inhibition	Peripheral tissues and immune tissues	T-cells	Inhibition of IL-17 was found to reduce mechanical allodynia and paw flinches in mouse models
Leukocyte Elastase Inhibition	Dorsal root ganglion	T-cells	Elane KO mice and SerpinA3N-treated mice showed significant reduction in the development of neuropathic pain
Fingolimod	N/A	CNS	Found to inhibit CNS T-cell infiltration
Glatiramer Acetate	CNS	T-cells	Found to convert pro- inflammatory T-cells into anti- inflammatory T-cells

Discussion 

As discussed above, the treatment of acute and chronic pain involves the use of NSAIDs, opioids, steroids, invasive procedures, and occasionally antidepressants. Although these treatment options are widely used, they are associated with serious adverse events. An unmet need exists for identifying both effective treatment options and more targeted approaches to chronic pain management. Developmental approaches focus on opioid receptor modulation and the subsequent analgesic effects without the opioid-associated adverse effects. Various approaches, which include those that are involved in altering the development, propagation, and/or CNS processing of pain, are being explored for pain modulation therapy. Although these developmental approaches have only been discussed in the context of pain modulation, there are additional implications of the above-mentioned therapies that can potentially broaden the use of these drugs clinically. Among all the aforementioned developmental approaches to chronic pain therapy discussed, four therapies hold the most promise in preclinical trials. These therapies include cytokine modulation, leukocyte elastase inhibitors, GPCR modulation, and sodium channel blockers. These therapies hold the most promise because, in addition to preclinical trials on hyperalgesia mice, these four therapies have also demonstrated preclinical and clinical trial success in a variety of pathologic conditions.

Pre-clinical research efforts have demonstrated that cytokine modulation, namely IL-1β and IL-17 receptors, results in a marked reduction in neuropathic allodynia [16,24]. As such, cytokine modulation may serve to selectively target a particular cytokine without interfering with intracellular signal transduction, as seen with GPCR modulation, and without altering the adaptive immune response, as seen with T-cell regulation. The inhibition of these cytokines and their subsequent effects throughout the body can potentially offer a system-wide reduction in the degree of tissue inflammation present and the consequent perception of pain signals. Meanwhile, there is a broader application of cytokine modulation, specifically IL-17 regulation. Based on experiments with mouse models that were xenografted with pancreatic cancer cells, it was found that IL-17R antagonism blocked the development of pancreatic cancer metastasis [28]. By utilizing an IL-17 antagonist for both chronic pain and cancer metastasis reduction. It can potentially reduce the amount of pain-modulating drugs cancer patients are taking, reduce potential drug-drug and drug-disease interactions, and provide both clinically therapeutic disease treatment and pain modulation. Although current studies have only been on mouse models and pancreatic cancer cell lineages, additional studies are underway in hopes of determining the extent of IL-17 inhibition in other forms of cancer [28].

In addition, it was found that treatment with leukocyte elastase inhibitors was beneficial in the treatment of respiratory disorders such as chronic obstructive pulmonary disease, acute lung injury, pulmonary fibrosis, and acute respiratory distress syndrome [29]. These findings are significant because they not only show additional applications for developmental therapy but also allow for the co-treatment of chronic pain and common pulmonary disorders, which commonly co-exist. The ability to provide dual therapy, reduce the adverse effects of opioids on respiratory depression, and still effectively treat pathology opens additional avenues for the clinical application of these medications. 

GPCR signal cascades produce their analgesic effects through an alteration in intracellular processes and signal cascades. Through these signal cascade alterations, GPCR regulation aims to increase the concentration of delta-opioid receptors on the neuronal plasma membrane [10-12]. Through increasing the concentration of delta-opioid receptors on the plasma membrane, this therapy has the potential to provide widespread pain modulation [10-12]. In addition, this holds potential for future research evaluating the combination of GPCR-modulating agents and currently used opioids, but at lower doses. Utilizing this potential co-treatment allows for an increase in the total number of DOR receptors present [10-12] and lowers the effective dose of exogenous opioids required to achieve a therapeutic effect. Furthermore, GPCR signal cascade modulation can potentially provide a longer-acting therapeutic benefit by altering an intracellular signal amplification cascade. 

Sodium channel blockers and granulocyte-colony stimulating factors are currently being used clinically as local anesthesia and for bone marrow hematopoiesis, respectively. In addition, research studies have demonstrated a significant reduction in pain tied to these agents. Sodium channel blockers target the primary perception of pain in the peripheral nervous system by preventing the propagation of a nerve impulse to the CNS [21]. Granulocyte-colony-stimulating factors aim to enhance neutrophil infiltration, a naturally occurring phenomenon upon injury, to potentially increase the local concentration of endogenous opioids produced by these cells [18]. Sodium channel blockers such as tricyclic antidepressants (TCAs) and anticonvulsants are occasionally repurposed to treat hyperalgesia-inducing states [30]. In a previously conducted study, patients who suffered from post-herpetic neuralgia and diabetic neuropathy demonstrated pain reduction when treated with TCAs [30]. This is one of the only treatment modalities that has been discussed that allows the bridging between mental illness and chronic pain. As painful conditions can potentially pose adverse mental health illnesses like depression, treatment with TCAs provides an avenue to treat the medical condition while also providing a method to prevent or reduce potential mental health ailments.

## Conclusions

Pain, as discussed in this article, is integral to survival. However, pain has the potential to evolve from acute to chronic. Although chronic pain has historically been treated with OTC medications such as NSAIDs, steroids, prescription opioids, and invasive procedures, there are numerous side effects tied to these treatments. Additionally, TCAs that have been repurposed to serve as an adjuvant to chronic pain therapy are not frequently prescribed due to their limited range of clinical use, limited data, and potential for adverse events. As a result of these side effects, alternative approaches to treating chronic pain are currently under investigation, such as delta opioid receptor agonists, LIM kinase inhibitors, RGS4 protein modulation, GPCR modulation, IL-1ßR modulation, granulocyte-colony stimulating factors, TIMP-1 and MMP modulation, sodium channel modulation, IL-17R modulation, leukocyte elastase inhibition, fingolimod (FTY720), and glatiramer acetate (GA). These therapies being explored are still in early development, with limited data on efficacy, long-term side effects, and tolerance available. While these agents appear to be promising, pain is processed at multiple cerebral levels. Therefore, it will be difficult to achieve sufficient pain reduction with a single pharmaceutical approach. As such, a potential future direction would be to utilize a cocktail therapy of two or more drugs to target the multilevel processing of pain signals. As this paper is a literature review and aims to provide a general narrative of the currently published literature on developmental opioid treatment modalities, there are limitations to this study. One such limitation is that of the primary articles used as references, only a summary of the results was provided rather than an in-depth discussion of the results. An additional limitation of this study is that most of these treatments are preclinical; thus, the efficacy and longevity of these treatments in patients have yet to be determined. Lastly, although no adverse events were observed in these developmental approaches, given that these therapies are preclinical and were conducted on mouse models, additional research is needed to further dispel potential side effects. All in all, chronic pain is a complex phenomenon, and additional research and literature are constantly developing and evolving in order to further explore this complex field of medicine and health. 
